# Direct Characterization of Halogen-Based Microplastics via Single-Event ICP-Mass Spectrometry in Negative-Ion Mode

**DOI:** 10.1021/acs.analchem.6c02321

**Published:** 2026-07-28

**Authors:** Antonio Bazo, Eduardo Bolea-Fernandez, Ana Rua-Ibarz, Martín Resano, Hamid Badiei, David Clases, Andrea Raab, Jörg Feldmann, Raquel Gonzalez de Vega

**Affiliations:** † Department of Analytical Chemistry, Aragon Institute of Engineering Research (I3A), 16765 University of Zaragoza, 50009 Zaragoza, Spain; ‡ PerkinElmer Scientific Canada ULC, Woodbridge L4L 8H1, Ontario, Canada; § NanoMicroLab, Institute of Chemistry, 27267 University of Graz, 8010 Graz, Austria; ∥ TESLA-Analytical Chemistry, Institute of Chemistry, University of Graz, 8010 Graz, Austria

## Abstract

Halogen-containing microplastics such as polytetrafluoroethylene (PTFE) and poly­(vinyl chloride) (PVC) are analytically relevant targets, yet their selective characterization by ICP-MS remains challenging, particularly for fluoropolymers due to the limited formation of F^+^ in conventional positive-ion mode. Here we introduce negative-ion single-event ICP-MS as a direct strategy for particle-resolved characterization of halogen-containing microplastics by monitoring F^–^ and Cl^–^ on a quadrupole ICP-MS without plasma modifiers or proxy-ion chemistry. PTFE and PVC particle standards were used as well-defined model systems, with scanning electron microscopy (SEM) confirming particle morphology and size distributions. Key acquisition and instrumental conditions governing event detectability were systematically optimized, enabling reliable transient detection at a dwell time of 100 μs. Using a conventional spray-chamber configuration, size detection limits of 1.18 μm (PTFE) and 0.73 μm (PVC) were achieved, improving to 0.68 μm (PTFE) and 0.45 μm (PVC) with a high-efficiency sample introduction system. Quantification strategies for negative-mode operation, including external calibration and transport-efficiency-based workflows, were further assessed. Overall, this work establishes negative-ion mode single-event ICP-MS as a direct platform for fluorine- and chlorine-selective microplastic detection and sizing, expanding the analytical scope of particle-resolved microplastic analysis beyond indirect fluorine detection or carbon-based approaches.

## Graphical abstract

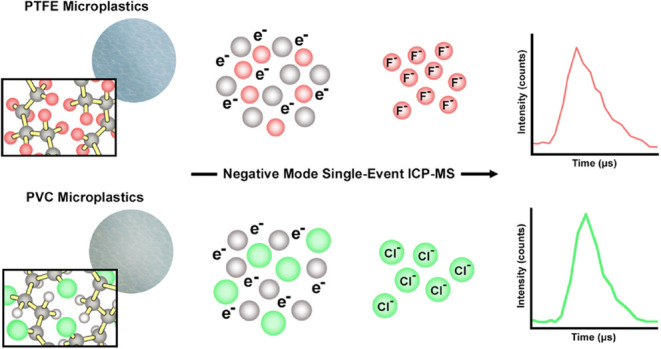

## Introduction

Microplastics are ubiquitous across natural and engineered systems, but their direct chemical characterization at the level of individual particles remains a major unresolved analytical challenge.^1–3^ Existing spectroscopic approaches can provide polymer-specific information, yet they often trade chemical specificity for throughput, struggle with smaller particle sizes, or are not readily suited to the direct analysis of heterogeneous particle suspensions.^4–6^ As a result, a critical capability remains missing: the rapid, event-resolved chemical discrimination of large numbers of individual microplastic particles in dispersed samples. Time-resolved mass spectrometric particle detection offers a promising route to close this gap as it enables high-throughput interrogation of individual particles while preserving chemically informative elemental signatures.^7^

In this context, single-event inductively coupled plasma-mass spectrometry (single-event ICP-MS) provides a powerful analytical tool in which discrete transient signals originating from individual particles can be used to derive particle number concentrations, size-related information, and chemical differentiation.^8–11^ Most ICP-MS-based strategies for microplastic analysis rely on carbon as a broadly applicable polymer marker;^12–14^ however, substantially greater chemical selectivity can be achieved by targeting heteroatoms intrinsic to specific polymer types. In this context, heteroatom- and label-based approaches have already demonstrated the value of single-event ICP-MS for particle counting and discrimination,^15,16^ yet its extension toward direct halogen-resolved characterization remains incomplete.

For fluorinated polymers in particular, this gap reflects a fundamental analytical limitation. Fluorine is difficult to determine by conventional positive-ion ICP-MS because its first ionization potential (17.42 eV) exceeds that of argon (15.76 eV), resulting in low ionization efficiency. As a consequence, fluorine analysis in ICP-MS has commonly relied on indirect strategies, most notably the detection of reaction products or proxy ions such as BaF^+^.^17–21^ These approaches have enabled important advances, but they also introduce an additional layer of chemistry between analyte and measurement and are not equivalent to direct event-resolved fluorine detection of individual particles. Establishing a direct route to fluorine-selective single-particle analysis would therefore address a long-standing measurement challenge and expand the analytical scope of single-event ICP-MS.

Chlorine-containing polymers represent a related but distinct case. Chlorine has already been explored as an informative target in ICP-MS-based microplastic analysis,^22^ and prior work has shown that chlorine signals can support selective polymer differentiation.^23^ Even so, a unified analytical strategy capable of directly targeting both fluorine- and chlorine-containing particles in an event-resolved manner would substantially broaden the chemical scope of elemental particle analysis. Such a strategy would be especially valuable since it would extend direct ICP-MS-based particle analysis to polymer types that remain difficult to access within existing workflows.

Negative-ion ICP-MS offers such an opportunity.^24–26^ Elements that readily form anions can, in principle, be monitored directly in negative mode, opening an alternative analytical window for selective particle characterization. Despite this conceptual promise, no studies have previously reported the use of negative-ion detection for single-event analysis, including applications to microplastic characterization. The central analytical question is therefore not only whether negative-ion mode detection is feasible, but whether it can provide sufficiently selective, sensitive, and quantitatively interpretable event signals for chemically distinct halogen-containing polymer particles.

In this work, we address this gap by establishing single-event negative-ion mode ICP-MS (single-event nICP-MS) for direct characterization of halogen-based microplastics. By targeting F^–^ and Cl^–^ signals, the method enables event-resolved detection of PTFE and PVC particles without relying on proxy-ion chemistry. Beyond demonstrating feasibility, we evaluate instrumental conditions that govern signal generation and transport, compare sample introduction strategies, and assess multiple quantification routes for converting transient event signal intensities into particle information. The aim is to define the analytical figures of merit, constraints, and opportunities of this measurement concept.

To establish the method under controlled conditions, commercially available PTFE and PVC particle standards were first characterized independently and then used as model systems for single-event nICP-MS. This design allows direct assessment of signal behavior, size-dependent response, and quantification performance for two analytically relevant halogen-containing polymer types. More broadly, the study positions negative-ion mode single-event ICP-MS as a new route for direct, chemically selective particle analysis and expands the range of accessible elements in particle-resolved ICP-MS.

## Experimental Section

### Reagents and Standards

For quantitative single-event nICP-MS, four PTFE particle standards (Polysciences, USA) with nominal diameters of 0.2 (SEM: 0.21 ± 0.05 μm), 1 (SEM: 0.24 ± 0.07 μm), 3 (SEM: 3.35 ± 1.31 μm) and 8 μm (amorphous shape), and three PVC particle suspensions (Lab261, USA) of 3 (2.62 ± 0.18 μm), 4 (3.69 ± 0.29 μm) and 5 (5.59 ± 0.51 μm) μm were analyzed. For PTFE, for which only an estimated particle size was provided by the manufacturer without associated uncertainty, particle size values and their uncertainties were determined by scanning electron microscopy (SEM). The SEM results revealed significant deviations from the expected sizes, particularly for the 1 and 8 μm PTFE particles. For PVC, particle diameters and corresponding uncertainties were provided directly by the manufacturer. All the suspensions were appropriately diluted in a 1.5% (v/v) Triton X-100 (Fisher Scientific, USA) solution prepared with ultrapure water (18.2 MΩ cm, Merck Millipore, Germany). For ionic calibration and optimization, ion-chromatography standards of 1 g L^–1^ F and Cl (Single-Element ICP-Standard-Solution RotiStar, Carl ROTH, Karlsruhe, Germany) were appropriately diluted in water.

### Instrumentation

All measurements were carried out using a NexION 2000 ICP-MS (PerkinElmer Inc., USA) configured for operation in negative-ion mode. To enable negative-ion mode detection, the detector and its associated power supply boards were replaced as described previously.^26^ The remaining ion-optical and mass-filter components, such as the quadrupole drivers and electrostatic lenses, are inherently bipolar and therefore can operate under both positive or negative voltages as needed. To minimize the contribution of fluorine-based contamination to the background signal, internal fluoropolymer material (e.g., tubing, seals, vacuum grease) was replaced with other material to the extent possible. The instrument interface was equipped with standard nickel skimmer and hyperskimmer cone (skimmer with a 0.6 mm aperture ID, and 1 mm for the hyperskimmer). To enhance sensitivity for fluorine and chlorine, a custom-modified sampler cone featuring a tapered tubular geometry was used. This design was improved by recent mechanistic evidence indicating that negative ion formation via electron capture or Penning ionization occurs predominantly postsupersonic expansion behind the sampler aperture. To optimize this process, the cone was engineered to maximize electron cosampling and extend analyte-electron residence times within the interface while maintaining a stable gas load. Specifically, the modified sampler was fabricated with a 4 mm long tapered tubular internal channel, featuring an entrance diameter of 2 mm and an exit diameter of 1 mm. This configuration preserves standard vacuum integrity while significantly improving ionization efficiency compared to conventional commercial geometries. A standard Fassel-type torch with a 1.5 mm injector ID was used. Two sample introduction configurations were evaluated: a traditional cyclonic spray chamber and a high-efficiency sample introduction system (Glass Expansion, Australia). Instrument settings and operating conditions are summarized in Table S1. For the characterization of the PTFE particles, SEM images were obtained using an Inspect F50 (FEI Company).

### Data Processing

Single event identification and integration was performed using the open-source python-based data processing platform “SPCal” (v1.4.9), using Poisson statistics to distinguish the single events from the ionic background.^27^ In addition, the Hyper Dimensional Image Processing software (HDIP v1.8.4) was used to identify the average peak profiles,^28^ and the OriginPro software (version 2021b, 9.85 OriginLab Corporation, USA) was used for further data processing and graphics preparation.

## Results and Discussion

### Optimization of the Instrument Settings and Operating Conditions

To optimize single-event ICP-MS measurements, instrument settings that influence both transport of entities and detection sensitivity must be considered. Among these, gas flow rates governing nebulization and aerosol transport are typically the most influential parameters, while RF power and related settings can also significantly impact sensitivity.^29^ In positive-ion mode ICP-MS (pICP-MS), such optimizations are commonly performed using ionic standard solutions, and settings are subsequently transferred to particle suspensions. However, due to differences in ion formation, where postplasma electron capture has been suggested as the most likely mechanism for negative-ion production,^30^ the suitability of using ionic standards for optimization rather than discrete particles needs to be evaluated for negative-mode single-event ICP-MS.

Prior to carrying out the above optimizations, appropriate quadrupole ion deflector (QID) voltages were selected, as negative-ion detection depends strongly on these parameters.^26^ The QID voltages were optimized for maximum sensitivity by monitoring a blank (0.14 M HNO_3_) and a solution containing 10 mg L^–1^ F and Cl. Negative voltages suppressed negative-ion transport into the quadrupole, while positive voltages enabled optimal detection of both ^19^F and ^35^Cl. Therefore, the optimized QID settings (100, 51, 37, and 19.5 V for attractor, box, entrance, and repellor, respectively) were applied throughout this work (Table S1).

Subsequently, the behavior of ionic standard solutions and particle suspensions was evaluated by monitoring a blank, a 10 mg L^–1^ F standard, and a 4 × 10^5^ particles mL^–1^ suspension of 3 μm PTFE particles at different nebulizer gas flow rates using a cyclonic spray chamber. The results for the ionic standard solution are shown in Figure 1A, where the best signal-to-background ratio (S/B) is obtained at a nebulizer gas flow rate of 0.7 L min^–1^. The signal intensity exceeded 450,000 cps for a 10 mg L^–1^ F solution, which was approximately 1 order of magnitude higher than that reported in a previous study.^26^ This difference can be attributed to the prototype stage of development of the negative-ion mode ICP-MS instrument and to the instrumental modifications implemented between the two studies. First, the use of a modified interface prolonged electron-analyte residence times and enhanced negative ion extraction efficiency far beyond the capabilities of the conventional commercial cones used in the previous work. Second, the instrument was equipped with a newly replaced electron multiplier detector, ensuring maximum detection efficiency and response. The combination of optimized interface fluid dynamics and pristine detector conditions fully accounts for the pronounced boost in sensitivity.

**Figure 1:**
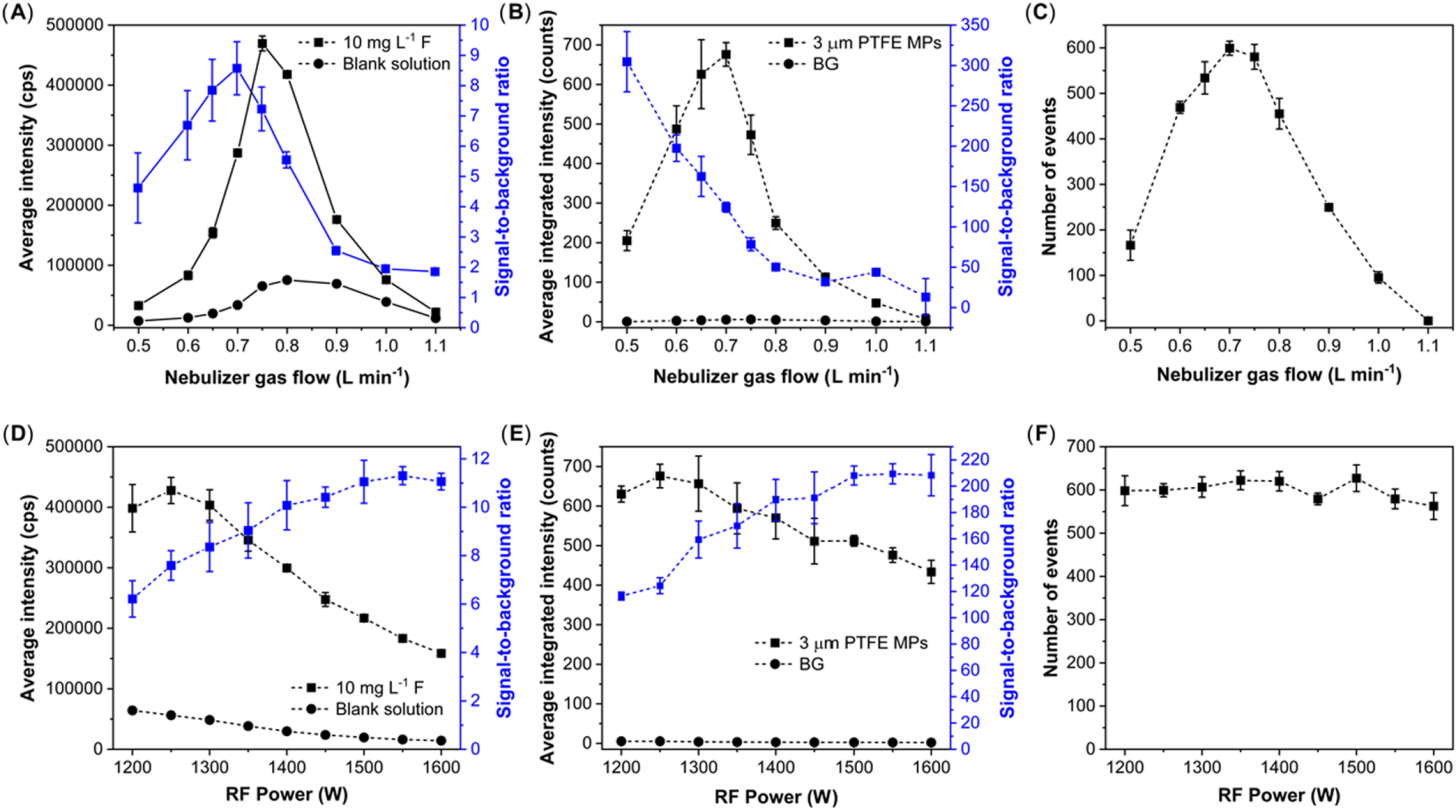
Effect of the nebulizer gas flow rate (A–C) and RF power (D–F) on the intensity and S/B ratio for a 10 mg L^–1^ ionic F and a 3 μm PTFE particles suspension, as well as for the number of events, respectively. Error bars represent the standard deviation of three replicate measurements (*n* = 3). Each measurement consisted of a 60 s acquisition time, using dwell times of 50 ms and 100 μs for the ionic and single-event measurements, respectively. The reported particle counts were obtained from a suspension with a particle number concentration of 4 × 10^5^ particles mL^–1^.

In the case of the PTFE particles (Figure 1B), the integrated intensity also reaches a maximum at this flow rate; however, the S/B ratio decreases steadily as the gas flow increases. This behavior can be explained by changes in the number of detected events (Figure 1C). The highest particle count is likewise observed at 0.7 L min^–1^, while at lower flow rates, where sensitivity is reduced, the smaller particles are not detected, leading to a bias in the apparent optimal S/B ratio.

A similar comparison was performed for the RF power where both the ionic standard solution (Figure 1D) and PTFE suspension (Figure 1E) exhibited the same trend, with no observable impact on the number of detected events (Figure 1F). Based on these results, an RF power of 1550 W provided the highest S/B ratio and was therefore selected for all subsequent measurements. Overall, nebulizer gas flow and RF power affected ionic standard and particle suspensions in a similar manner, consistent with previous observations in pICP-MS.^11^

The optimizations described above were carried out using a traditional cyclonic-type spray chamber. However, such introduction systems are designed to filter out large droplets from the nebulized aerosol. This low-pass filtering effect of the spray chamber strongly discriminates against low micrometer-sized particles, particularly those larger than 5 μm, significantly compromising transport efficiency (TE) and, consequently, the accuracy of the measurement results.^31^ To overcome this limitation, high-efficiency sample introduction systems are often employed when introducing large discrete entities, such as cells. In this work, such a system was used to enhance micrometer-scale PTFE and PVC particle introduction. This setup operates by combining two Ar flows: a nebulizer gas and a makeup gas.^32^ Therefore, an additional optimization was required to maximize instrument sensitivity for both ^19^F and ^35^Cl using a 10 mg L^–1^ ionic standard solution. However, it should be noted that these sample introduction systems were designed to maximize the transport of micrometer-scale entities and may therefore behave differently from conventional spray chambers when introducing ionic standard solutions. In these systems, sensitivity is primarily governed by the total gas flow, defined as the sum of the nebulizer and makeup gas flows. Accordingly, the nebulizer gas flow rate was fixed at 0.5 L min^–1^, while the makeup gas flow was adjusted stepwise to assess the sensitivity as a function of total gas flow.^33^ The results are shown in Figure 2, where the highest S/B ratio for F is observed at a makeup gas flow of 0.7 L min^–1^ (Figure 2A), while the optimal value for Cl is 0.8 L min^–1^ (Figure 2B); total gas flow rates of 1.2 and 1.3 L min^–1^ for F and Cl, respectively.

**Figure 2:**
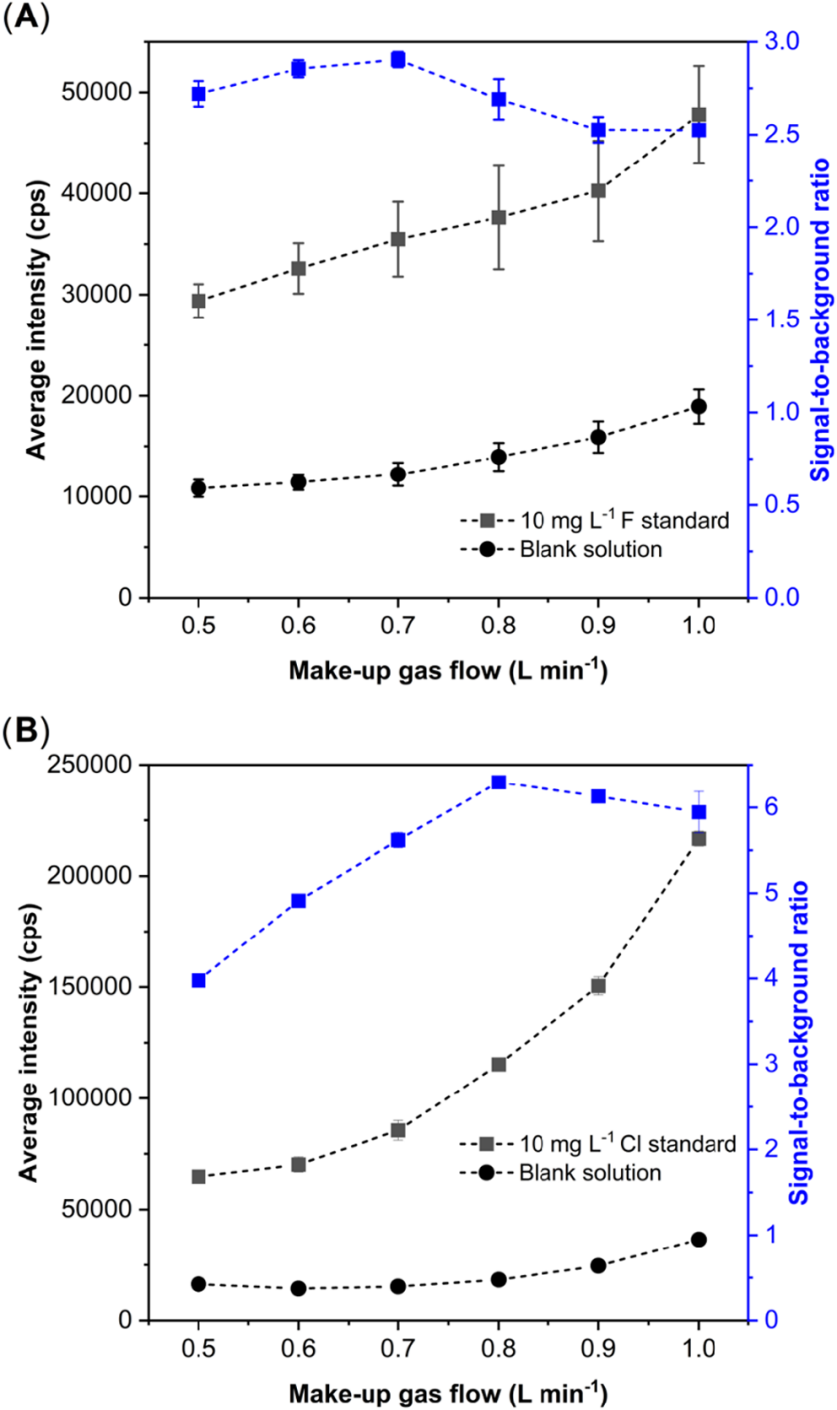
Effect of the makeup gas flow rate on the signal intensity and S/B ratio for 10 mg L^–1^ standard solutions of F (A) and Cl (B). Error bars represent the standard deviation of three replicate measurements (*n* = 3). Each measurement consisted of a 60 s acquisition time using a dwell time of 50 ms. The nebulizer gas flow rate and RF power were fixed at 0.5 L min^–1^ and 1550 W, respectively.

However, even under optimized conditions, the sensitivity obtained with the high-efficiency sample introduction system was lower than that achieved using the cyclonic spray chamber. Differences in transport efficiency and sample uptake rate could not fully account for this behavior, which is most likely related to a design that favors the introduction of discrete entities rather than ionic standard solutions. In addition, the minimum sample uptake rate available in this work (50 μL min^–1^) was suboptimal for efficient transport of the nebulized aerosol, leading to sensitivity losses due to aerosol deposition on the spray chamber walls. This interpretation is further supported by the different responses of the two sample introduction systems to changes in nebulizer gas flow. While the cyclonic spray chamber exhibited a clear maximum in signal intensity, the high-efficiency system showed a continuous increase in signal intensity with increasing nebulizer gas flow.

These observations suggest that optimal conditions for the introduction of ionic standard solutions were not reached with the high-efficiency setup, even though the selected conditions enabled efficient transport of micrometer-scale particulate material. Self-evidently, potential differences in the fundamental mechanisms of anion formation between ionic standard solutions and microparticulate materials cannot be ruled out. Such differences may have contributed, at least in part, to some of the observations discussed above. Further work should therefore focus on improving our understanding of the mechanisms governing anion formation and their potential dependence on the analyte nature.

Nevertheless, the optimization procedure allowed identification of operating conditions that provided the best compromise between instrument sensitivity and signal-to-background ratio. Based on this criteria, makeup gas flow rates of 0.7 and 0.8 L min^–1^ were selected for the subsequent analysis of F- and Cl-containing particles, respectively.

In addition to optimizing the instrument settings for maximum sensitivity, the characteristic fast acquisition rates of single-event measurements often require optimization of the dwell time, which is especially important for determinations affected by relatively high background signals. In this case, the measurement of F is influenced by potential contamination, the occurrence of spectral interferences (e.g., ^18^O^1^H^–^ at *m*/*z* 19), or electrons reaching the detector, as described elsewhere.^34^ The higher uncertainty observed for F relative to Cl in Figure 2 is most likely attributable to these factors.

To assess the effect of the dwell time, 3 μm PTFE particles were monitored at dwell times of 20, 50, 100, 200, 500, 1000, and 3000 μs.^35^ The results are shown in Figure S1. As can be seen, no significant differences in the average integrated intensities were observed across the different dwell times; however, higher uncertainties were observed at the extreme dwell time values. This is due to the fact that the shortest dwell times distribute the event signals over a larger number of data points, producing very low raw counts that are indistinguishable from the background, and are therefore not considered during event integration. On the other hand, longer dwell times include a larger contribution from the background signal. Based on these results, a dwell time of 100 μs was selected for all subsequent single-event nICP-MS analysis, exhibiting the lowest measurement uncertainty.

### Figures of Merit

Following optimization of the instrument settings and operating conditions for single-event nICP-MS analysis, Figure 3 shows the time-resolved signals, the frequency versus integrated intensity distributions, and the average signal events for 3 μm PTFE (Figure 3A) and 3 μm PVC (Figure 3B) particles. As shown, single-event nICP-MS produces the expected time-resolved signal comprising discrete particle events distributed across the acquisition period, which in turn generates the corresponding frequency–integrated intensity distribution. These results demonstrate that negative-ion mode can be effectively implemented for single-event ICP-MS at microsecond dwell times,^36^ enabling the characterization of elements prone to anion formation or that exhibit difficulties forming positive ions. In this context, it may represent a significant advancement for the selective analysis of micrometer-sized halogen-based polymeric particles (i.e., microplastics), allowing both detection and size characterization through direct monitoring of F^–^ and Cl^–^ anions.

**Figure 3:**
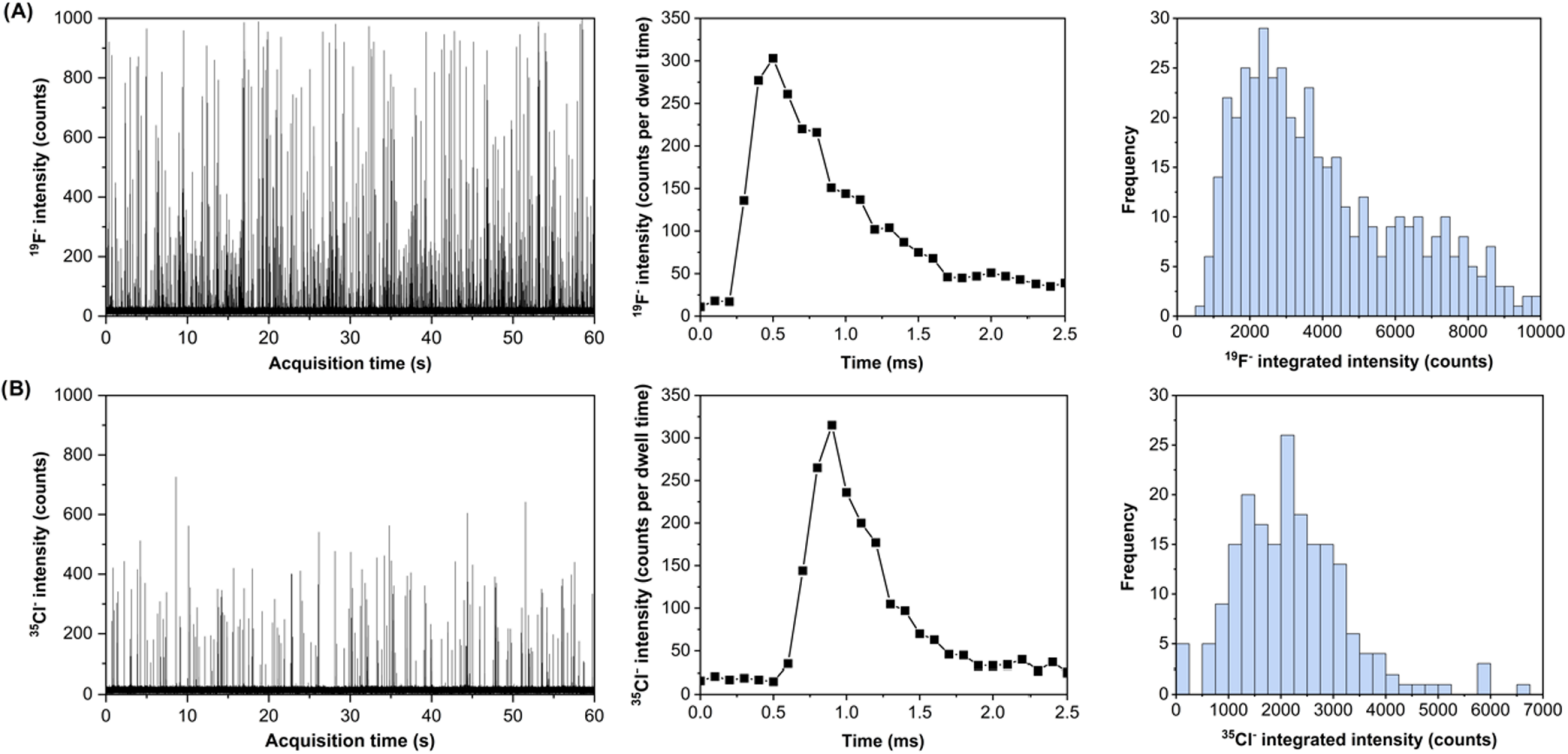
Single-event nICP-MS time-resolved signal, average duration of the individual events, and frequency versus integrated intensity distribution for a 3 μm PTFE (A) and 3 μm PVC particles (B).

The average peak profiles of the individual events appear broader, with an asymmetric right-hand tail, than those typically observed in positive mode (average duration for individual events = 1680 ± 547 μs and 1540 ± 351 μs for 3 μm PTFE and 3 μm PVC particles, respectively). The broader and asymmetric single-event peak profiles observed in negative-ion mode likely reflect fundamental differences in anion formation compared to conventional positive-ion ICP-MS. While positive ions are generated directly within the high-temperature plasma, negative ions are predominantly formed via postplasma electron attachment, occurring within the expanding interface region and early ion optics. As this process is spatially distributed rather than confined to a localized plasma zone, anion formation is expected to proceed over an extended region, leading to increased temporal dispersion of the resulting ion cloud. In addition, electron attachment inherently involves collisional processes, which have previously been shown to induce peak broadening in single-event ICP-MS under collision/reaction conditions.^28^ Together, these effects provide a plausible explanation for the longer event durations and asymmetric peak shapes (possibly a Boltzmann distribution) observed in negative-ion single-event measurements.^37^

To further assess the potential of single-event nICP-MS, the detection capabilities were evaluated and compared with those of single-event pICP-MS. The mass detection limits (LoD_mass_) were calculated according to eq 1, where s_bl_ is the standard deviation of the blank in counts (4.76 counts for ^19^F^–^, and 1.42 counts for ^35^Cl^–^), w is the expected event duration for a particle at the LoD (conventionally approximated as 500 μs), k is the integrated sensitivity (0.110 counts fg^–1^ for ^19^F^–^, and 0.236 counts fg^–1^ for ^35^Cl^–^) determined from a calibration curve constructed with analyte-containing particles, and t_dwell_ is the dwell time in μs. Assuming sphericity, the size detection limits (LoD_size_) were then determined for particles of known density (ρ, in fg μm^–3^) and elemental composition (i.e., the molar mass of the particle, M_part_, and the molar mass of the analyte within each entity, M_an_), as shown in eq 2.^38,39^

1 $${\mathrm{LoD}}_{\mathrm{mass}}=\frac{3s_{\mathrm{bl}}w}{2kt_{\mathrm{dwell}}}$$

2 $${\mathrm{LoD}}_{\mathrm{size}}=\sqrt[{3}]{\frac{{\mathrm{LoD}}_{\mathrm{mass}}6}{\mathrm{\pi \rho}}\frac{M_{\mathrm{part}}}{M_{\mathrm{an}}}}$$

Using these equations, the LoD_size_ obtained with the cyclonic spray chamber was estimated to be of 1.18 μm for PTFE and 0.73 μm for PVC. As shown in Table 1, these values are already competitive when compared to those reported for positive-ion mode measurements. The use of the high-efficiency sample introduction system enabled additional improvements, yielding LoD_size_ values as low as 0.68 μm for PTFE and 0.45 μm for PVC.

**Table 1:** Comparison of the LoD_size_ Values Reported in Literature for the Size Characterization of Halogenated Polymer-Based Microparticles (Microplastics) by Monitoring Different Target Nuclides

halogenated particle	mass analyzer	introduction system	species	LoD_size_, μm	reference
PTFE	Q (N_2_ plasma)	scott-type	^12^C^+^	∼1.0	^40^
PTFE	Q (MS)	cyclonic	^157^BaF^+^	0.93	^17^
PTFE	Q (MS/MS)	scott-type	^157^BaF^+^	1.1	^18^
PTFE	Q (MS)	cyclonic–high-efficiency	^19^F^–^	1.18–0.68	this work
PVC	Q (MS/MS)	laser ablation	^13^C^+^	1.2	^41^
PVC	TOF	high-efficiency	^35^Cl^+^	3.0	^22^
PVC	TOF	cyclonic	^12^C^+^	1.4	^23^
PVC	TOF	cyclonic	^37^ClH_2_^+^	1.3	^23^
PVC	Q (MS)	cyclonic–high-efficiency	^35^Cl^–^	0 73–0.45	this work

It should be noted that the accuracy of the estimation of these parameters is intimately affected by the selection of the reference standards. In this context, while the PVC particles were spherical and monodisperse, the PTFE standard was amorphous and polydisperse, which may affect the determined LOD_size_. However, this very same standard (or one of similar quality) was used in the different works collected in Table 1, so no biases are expected for the comparison. Moreover, an event duration of 500 μs was assumed, although this value likely underestimates the actual transit time of a particle at the LoD and may therefore influence the estimated size detection limits. However, accurately determining this signal duration is highly challenging, and the conventionally adopted value of 500 μs was retained to enable comparison with previous studies. While this approach was considered fit for purpose in the present work, the use of peak height may provide a more realistic basis for LoD estimation,^39^ as demonstrated previously, and deserves further consideration in future studies. Nevertheless, these figures of merit demonstrate that single-event nICP-MS holds promise for the direct analysis of micrometer to submicrometer sized halogen-based polymeric particles.

### Characterization of F- and Cl-Containing Polymer-Based Microparticles

To convert the integrated intensity of each event into the corresponding analyte mass per particle and subsequently calculate the particle size and size distribution of a particle population, appropriate calibration strategies must be established. Single-event pICP-MS often relies on metallic nanoparticle (NP) standards, such as AuNPs, to determine a correction factor, often referred to as TE_ionic_, that enables calibration with ionic standards of the target analyte.^42^ However, these NP standards do not form anions suitable for detection in single-event nICP-MS, thus necessitating alternative calibration strategies compatible with negative-mode operation.

In this context, existing strategies developed for single-event pICP-MS were evaluated for their potential adaptation to negative-ion mode, using PTFE and PVC particles as model F- and Cl-containing particles. The approaches assessed include methods that are independent of TE determination (i.e., external calibration) as well as those that depend on TE (i.e., particle size, particle frequency, and waste collection).^43^ The results obtained for each strategy using both types of sample introduction systems are summarized in Figure 4.

**Figure 4:**
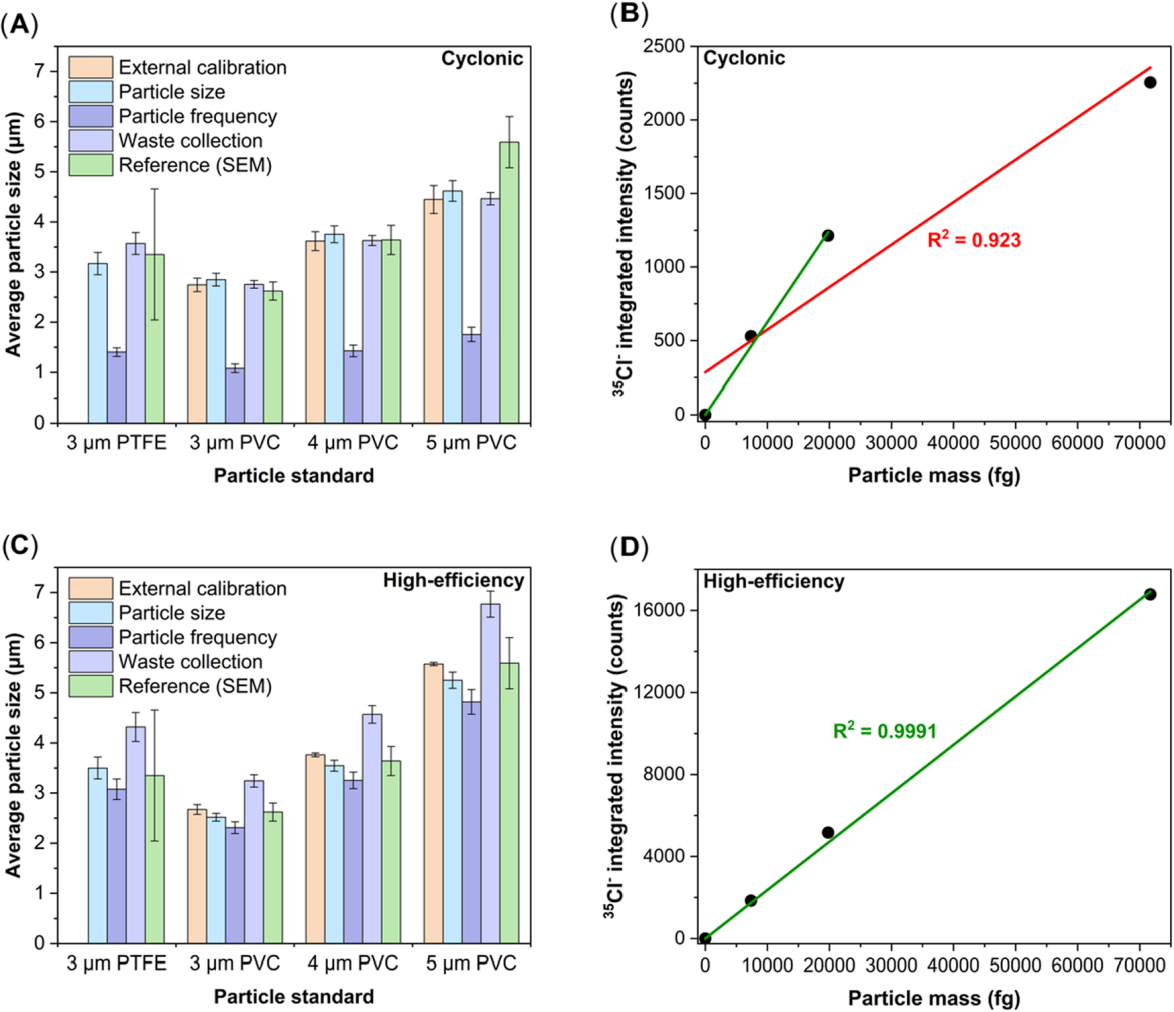
Comparison of the results obtained for the characterization of PTFE and PVC particle standards using different quantification strategies (A, C) and their corresponding calibration curves (B, D) obtained with the cyclonic spray chamber and the high-efficiency spray chamber for sample introduction, respectively. The error bars of the sizes determined by single-event nICP-MS represent the combined uncertainty, calculated as discussed elsewhere.^43^ The uncertainty in the SEM results corresponds to the standard deviation (*n* ≈ 200). The green solid lines represent the linear range of the external calibration, while the red solid line illustrates the loss of linearity for the cyclonic spray chamber when fitting the calibration curve including the 5 μm PVC particle standard.

First, external calibration is performed using particle standards with known analyte mass, typically derived from the particle size, density, and chemical composition (assuming spherical shape). Provided an adequate mass value is available or can be derived, the integrated intensities of all events can be directly interpolated into the calibration curve to determine the analyte mass. This method has been shown to be more accurate and direct than other strategies for quantitative single-event pICP-MS analysis.^44^ However, its applicability is limited by the availability of suitable particle standards for calibration.

In this work, while appropriate PVC particle standards were available for Cl, F determination relied only on the 3 μm (3.35 ± 1.31 μm; Figure 5C) PTFE standard, which could be used for external calibration of other samples but is not meaningful for calibrating itself. The 0.2 μm (0.21 ± 0.05 μm; Figure 5A) and 1 μm (0.24 ± 0.07 μm; Figure 5B) PTFE standards fell below the detection limit (0.68 μm). In addition, the 8 μm PTFE material consisted of large fibers that clogged the nebulizer (Figure 5D). As a result, external calibration could only be applied to the size determination of PVC particles, yielding highly accurate and precise results for all standards except the 5 μm PVC particles introduced using a cyclonic spray chamber (Figure 4A). This bias in the sizing of these particles can be attributed to the discrimination of the larger particle fraction occurring in low-efficiency sample introduction systems, which are not suitable for introducing micrometer-scale particles. Under these conditions, only the smaller fraction of the particle population was efficiently transported, resulting in fewer recorded events, an underestimation of the integrated intensities, and therefore an underestimation of mass and size. This effect is clearly reflected in the loss of linearity shown in Figure 4B (*R* ^2^ = 0.923 when the data point for the larger 5 μm PVC particles is included, and 0.997 when it is excluded). In contrast, the use of the high-efficiency sample introduction system (Figure 4C) maximized transport for this particle-size range (3–5 μm; *R* ^2^ = 0.9991), extending the linear range and improving method accuracy, as demonstrated in Figure 4D.

**Figure 5:**
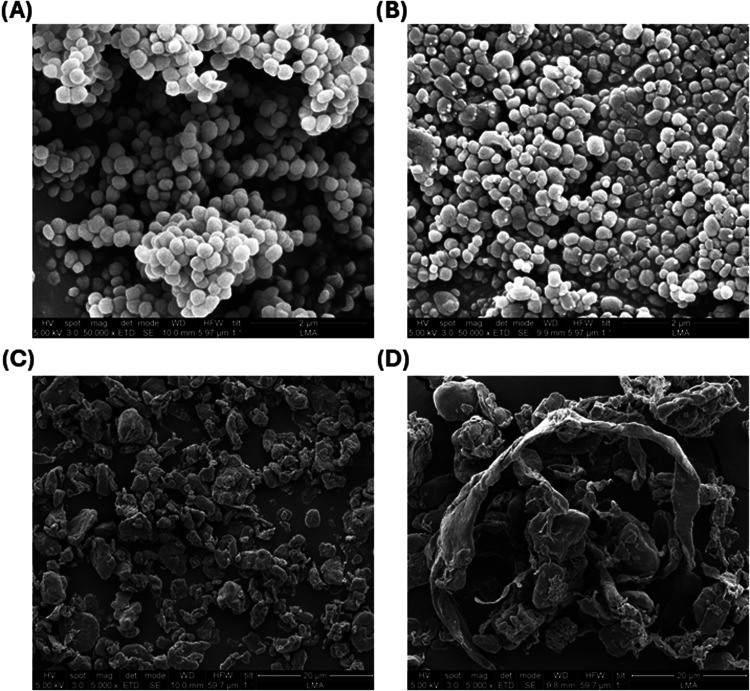
Representative SEM images of the 0.2 (A), 1 (B), 3 (C), and 8 (D) μm PTFE particles analyzed in this work.

In contrast to the external calibration approach, TE-dependent methods rely on determining the TE constant to convert the integrated intensity of each event (I_part_, counts) into the corresponding analyte mass (m_part_, fg), according to eq 3, where Q_neb_ is the sample flow rate in mL min^–1^, t_dwell_ is the dwell time in seconds, and S_ion_ is the analyte sensitivity in counts mL fg^–1^. As indicated above, three main strategies have typically been used to calculate this correction factor, namely the particle size, particle frequency, and waste collection approaches, and their suitability for the size characterization of F- and Cl-based polymeric particles was evaluated.

3 $$m_{\mathrm{part}}=\frac{I_{\mathrm{part}}t_{\mathrm{dwell}}Q_{\mathrm{neb}}\mathrm{TE}M_{\mathrm{part}}}{S_{\mathrm{ion}}M_{\mathrm{an}}60}$$

The particle size method generally determines the TE by monitoring nanoparticle standards of well-defined size. Unlike external calibration, this method relies on particulate material that does not necessarily need to contain the target analyte in its chemical composition. The TE can be calculated using eq 4, where S_part_ is the sensitivity of the particle standard in counts fg^–1^. Because metallic nanoparticle standards commonly used in single-event pICP-MS (e.g., AuNPs) are unsuitable for negative-ion mode, PVC standards were used to determine the TE_part. size_ for PTFE, and *vice versa*. The TE values obtained were 7.56 ± 0.84% and 8.94 ± 0.98% for the cyclonic spray chamber (using PVC and PTFE, respectively), and 32.7 ± 1.8% and 28.7 ± 1.4% for the high-efficiency sample introduction system (using PVC and PTFE, respectively). The slight differences between the TEs determined using PVC or PTFE standards can be attributed to some degree of polydispersity associated with these materials, whereas the differences between chambers are not only due to differences in transport itself, but also to the fact that the cyclonic spray chamber is designed to remove the larger nebulized droplets, leading to intensity histograms that are slightly biased toward lower values. As shown in Figure 4, the accuracy of this approach was comparable to that of external calibration, with significant deviations only observed for the 5 μm PVC particles introduced using the cyclonic spray chamber. This bias results from the less favorable transport of larger particles in low-efficiency sample introduction systems. The main advantage of this strategy is that it does not require standards containing the target analyte.

4 $${\mathrm{TE}}_{\mathrm{part}.\mathrm{size}}=\frac{M_{\mathrm{an}}S_{\mathrm{ion}}60}{M_{\mathrm{part}}Q_{\mathrm{neb}}S_{\mathrm{part}.\mathrm{std}}t_{\mathrm{dwell}}}$$

It should be noted that, somewhat counterintuitively, the TE obtained using the particle size method does not directly reflect the transport of ionic solutions or suspended particles. Instead, it serves as a proportionality constant that correlates the intensity of single events with their corresponding particle mass through an ionic calibration curve. By contrast, the TE determined using the particle frequency method is a direct indicator of how efficiently particles are transported to the plasma. For this approach, TE_part. freq_ is also traditionally determined using nanoparticle standards, which in this case must be well-characterized in terms of their particle number concentration (PNC). The correction factor is calculated by monitoring the number of events per minute of analysis (N, min^–1^), according to eq 5. Successful application of the particle frequency method for size determination assumes identical transport behavior for ionic standard solutions and particulate material. While this assumption holds reasonably well for nanoparticles (sizes <100 nm), relatively large entities, such as those in the micrometer size range, exhibit less favorable transport,^45,46^ even when high-efficiency sample introduction systems are used. This leads to an underestimated TE for sizing, although the method remains suitable for determining the PNC of micrometer-sized particles. This explains the significant deviations observed for all particle sizes when using the cyclonic spray chamber (TE_part. freq_ = 0.49 ± 0.11%) and the slight underestimation obtained with the high-efficiency sample introduction system (TE_part. freq_ = 22.2 ± 1.1%).

5 $${\mathrm{TE}}_{\mathrm{part}.\mathrm{freq}}=\frac{N}{\mathrm{PNC}Q_{\mathrm{neb}}}$$

In addition to the particle size and particle frequency approaches, the waste collection method can serve as an alternative when suitable particle standards are unavailable. Here, TE_waste_ (eq 6) is determined gravimetrically using an ionic standard solution by introducing a known mass (m_int_) and weighing the fraction not transported into the plasma collected from the waste channel (m_waste_). In contrast to the previous approaches, this method proved unsuitable for the high-efficiency sample introduction system. Under these conditions, a substantial portion of the untransported liquid was not recovered in the waste outlet, most likely due to retention and evaporation on the spray chamber walls. This leads to an overestimation of transport (TE_waste_ = 61.5 ± 5.4%), resulting in significant deviations for both PTFE and PVC particles. Conversely, such drying losses are nearly negligible in the cyclonic spray chamber (TE = 8.08 ± 0.28%). Accordingly, particle sizes derived from this approach agreed well with the SEM reference values, with the exception of the 5 μm PVC particles, where transport limitations for larger particles, as discussed above, resulted in biased sizing.

6 $${\mathrm{TE}}_{\mathrm{waste}}=\frac{m_{\mathrm{int}}-m_{\mathrm{waste}}}{m_{\mathrm{int}}}$$

Overall, the different strategies commonly applied to single-event pICP-MS analysis could also be implemented in negative-ion mode. However, their applicability depends on the availability of suitable particle standards containing the target analyte or on the extent to which particle size and number concentration are adequately characterized.

## Conclusions and Future Perspectives

In this work, we demonstrate, for the first time, the feasibility and analytical potential of negative-ion mode single-event ICP-MS for the characterization of halogen-based polymeric particles (microplastics). Following the adaptation of the ICP-MS platform for negative-ion mode, optimized conditions enabled the acquisition of time-resolved signals suitable for single-entity analysis, allowing the use of ionic standards rather than discrete entities for optimization. Halogen-based polymeric particles, which are traditionally challenging to analyze directly in positive-ion mode, were successfully characterized, with particular emphasis on the size determination of PTFE particles through direct monitoring of F^–^ ions, thereby avoiding the need to rely on F-based molecular ions as indirect proxies. Although the peak durations of individual events were found to be longer than those typically observed in positive-ion mode, implementation of a high-efficiency sample introduction system provided size detection limits of 0.68 μm for PTFE and 0.45 μm for PVC. These results significantly improved those previously obtained by positive-ion mode operation and highlight the potential of negative-ion mode single-event ICP-MS for sizing and characterization of microparticulate material containing elements prone to anion formation. Calibration and quantification strategies established for positive-ion mode single-event ICP-MS were also applicable under negative-ion mode operation. However, the lack of sufficiently monodisperse, well-characterized particle standards in terms of both size and number concentration remains a limiting factor and can compromise measurement accuracy.

Overall, negative-ion mode single-event ICP-MS provides a direct route for halogen-specific particle characterization, including fluorine-resolved microplastic analysis without proxy-ion strategies. While positive-ion mode ICP-MS is widely established, negative-ion mode is still in an early stage of development. However, the prototype nature of the instrumentation leaves room for further improvements in sensitivity and F background reduction, which would substantially enhance detection limits. In this regard, the determination of per- and polyfluoroalkyl substances (PFAS), for which compound-specific regulations are becoming increasingly stringent, may also benefit from negative-ion mode operation, opening new avenues for ICP-MS analysis. Furthermore, an interchangeable ICP-MS platform capable of operating in both positive and negative modes would provide a more versatile route to exploit complementary cation and anion monitoring, further expanding the analytical scope of single-event ICP-MS.

## Supplementary Material



## Data Availability

Data will be available through Zenodo.

## Notes

Instrument settings and operating conditions (Table S1); evaluation of dwell time effects on the integrated intensity of 3 μm PTFE particles (Figure S1) (PDF)
