# Supplementary material for: Direct Characterization of Halogen-Based Microplastics via Single-Event ICP-Mass Spectrometry in Negative-Ion Mode
Source: Anal Chem. 2026 Jul 28;98(31):22977–87. doi: 10.1021/acs.analchem.6c02321 (PMC13470986; doi:10.1021/acs.analchem.6c02321)
Supplement: Supplementary file 1 [file ac6c02321_si_001.pdf]

Supporting Information

Direct characterization of halogen-based microplastics via single-event ICP-mass spectrometry in negative-ion mode

Antonio Bazo[1], Eduardo Bolea-Fernandez[1], Ana Rua-Ibarz[1], Martín Resano[1], Hamid Badiei[2], David Clases[3], Andrea Raab[4], Jörg Feldmann[4], Raquel Gonzalez de Vega[4]*

[1]Department of Analytical Chemistry, Aragon Institute of Engineering Research (I3A), University of Zaragoza, 50009 Zaragoza, Spain

[2]PerkinElmer Scientific Canada ULC, Woodbridge, L4L 8H1 Ontario, Canada

[3]NanoMicroLab, Institute of Chemistry, University of Graz, 8010 Graz, Austria

[4]TESLA-Analytical Chemistry, Institute of Chemistry, University of Graz, 8010 Graz, Austria

Corresponding author: Raquel.gonzalez-de-vega@uni-graz.at

Table of contents for Supporting Information

Table S1. Instrument settings and operating conditions.

Figure S1. Effect of the dwell time on the average integrated intensity for 3 µm PTFE particles. Error bars represent the standard deviation of three replicate measurements (n = 3). Each measurement consisted of a 60 s acquisition time using a dwell time of 100 µs. The reported particle counts were obtained from a suspension with a particle number concentration of $4x10^5$ particles mL^{-1}.

Table S1. Instrument settings and operating conditions.

	Cyclonic	High-efficiency
**Measurement conditions**		
RF Power, W	1550	
Nebulizer gas flow, L min^{-1}	0.7	0.5
Make-up gas flow, L min^{-1}	-	0.7 (F), 0.8 (Cl)
Auxiliary gas flow, L min^{-1}	0.85	
Cell rod off-set, V	+9	
Quadrupole rod off-set, V	+19.5	
Cell entrance/exit, V	+18	
QID attractor, V	+100	
QID box, V	+51	
QID entrance, V	+37	
QID repellor, V	+19.5	
Detector, V	+750 (first dynode), +3100 (last dynode)	
Detector gain, V	2350	
Discriminator	10	
**Instrumental setup**		
Sample introduction system	Cyclonic spray chamber<br>Meinhard glass nebulizer	High-efficiency spray chamber<br>Single-cell nebulizer<br>(Glass Expansion, Germany)
Introduction system	Peristaltic pump	Syringe pump
Uptake rate, mL min^{-1}	0.35 – 0.37	0.05

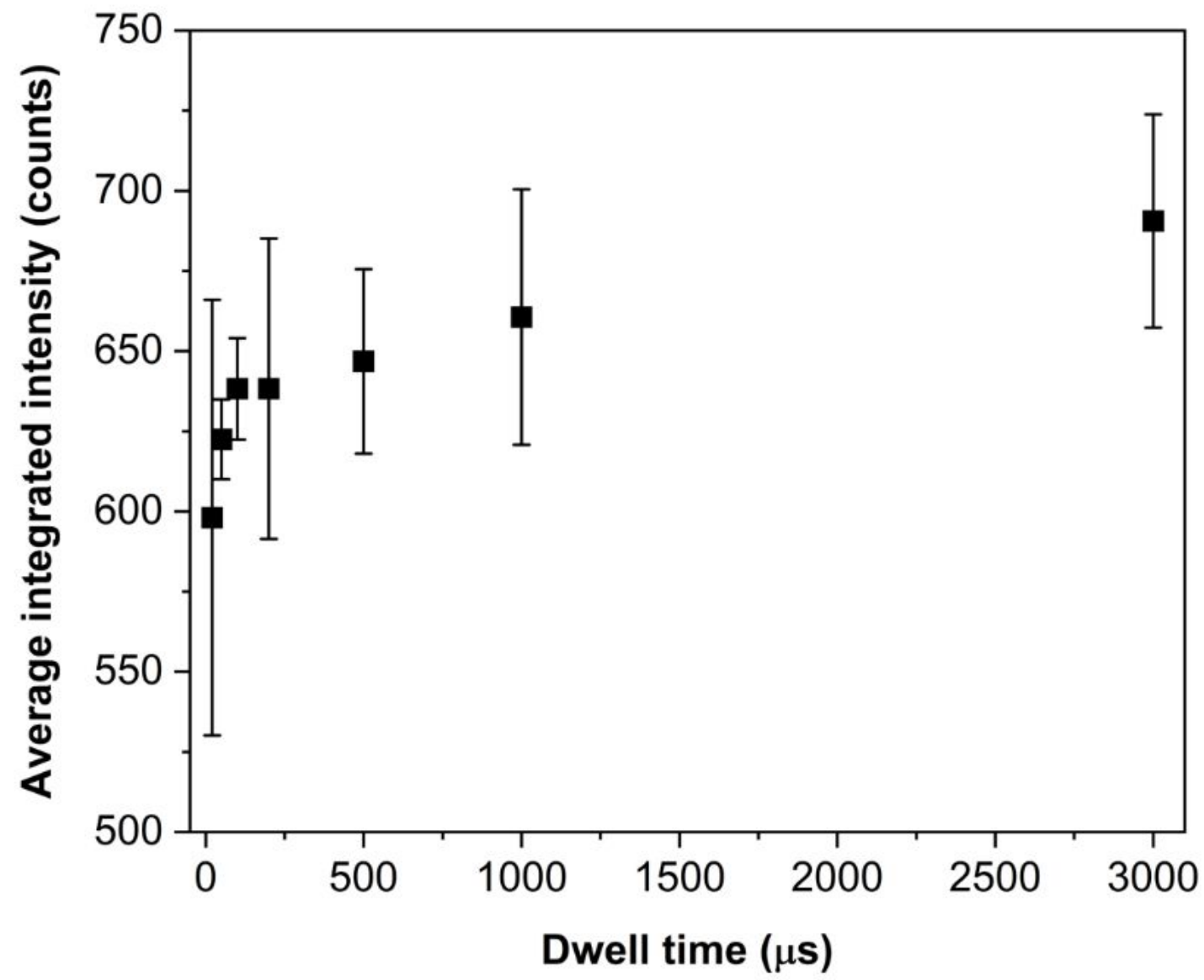

Figure S1. Effect of the dwell time on the average integrated intensity for 3 μm PTFE particles. Error bars represent the standard deviation of three replicate measurements (n = 3). Each measurement consisted of a 60 s acquisition time using a dwell time of 100 μs. The reported particle counts were obtained from a suspension with a particle number concentration of $4x10^5$ particles mL^{-1}.
